# Supplementary material for: Plasmid2MC: efficient cell-free generation of high-purity minicircle DNA for genome editing in mammalian cells
Source: Commun Biol. 2025 Dec 16;8:1778. doi: 10.1038/s42003-025-09157-7 (PMC12708848; doi:10.1038/s42003-025-09157-7)
Supplement: Supplementary file 2 — Description of Additional Supplementary Materials [file 42003_2025_9157_MOESM2_ESM.pdf]

Description of Additional Supplementary Files

File name: Supplementary Data 1

Description: 1. Recombination Yields Table.xlsx. 2. Base Editing Efficiency Sanger-EditR Results.xlsx. 3. Base Editing plasmid, mcDNA and gDNA CV sequences. 4. HITI plasmid, mcDNA and gDNA CV sequences. 5. Sequenced Plasmidsaurus recombined mcDNA.
